# Antibody-Mediated LILRB2-Receptor Antagonism Induces Human Myeloid-Derived Suppressor Cells to Kill *Mycobacterium tuberculosis*

**DOI:** 10.3389/fimmu.2022.865503

**Published:** 2022-06-10

**Authors:** Vipul K. Singh, Arshad Khan, Yitian Xu, Sunny Mai, Licheng Zhang, Abhishek Mishra, Blanca I. Restrepo, Ping-Ying Pan, Shu-Hsia Chen, Chinnaswamy Jagannath

**Affiliations:** ^1^ Department of Pathology and Genomic Medicine, Houston Methodist Research Institute, Houston, TX, United States; ^2^ Center for Immunotherapy Research and Cancer Center, Weill Cornell Medicine, Houston Methodist Research Institute, Houston, TX, United States; ^3^ School of Public Health at Brownsville, University of Texas Health Science Center Houston, Brownsville, TX, United States; ^4^ South Texas Diabetes and Obesity Institute, University of Texas Rio Grande Valley, Edinburg, TX, United States

**Keywords:** tuberculosis, MDSC (myeloid-derived suppressor cell), LILRB, monoclonal antibody, Mycobacterium

## Abstract

Tuberculosis is a leading cause of death in mankind due to infectious agents, and *Mycobacterium tuberculosis* (Mtb) infects and survives in macrophages (MФs). Although MФs are a major niche, myeloid-derived suppressor cells (MDSCs) are an alternative site for pathogen persistence. Both MФs and MDSCs express varying levels of leukocyte immunoglobulin-like receptor B (LILRB), which regulate the myeloid cell suppressive function. Herein, we demonstrate that antagonism of LILRB2 by a monoclonal antibody (mab) induced a switch of human MDSCs towards an M1-macrophage phenotype, increasing the killing of intracellular Mtb. Mab-mediated antagonism of LILRB2 alone and its combination with a pharmacological blockade of SHP1/2 phosphatase increased proinflammatory cytokine responses and phosphorylation of ERK1/2, p38 MAPK, and NF-kB in Mtb-infected MDSCs. LILRB2 antagonism also upregulated anti-mycobacterial *iNOS* gene expression and an increase in both nitric oxide and reactive oxygen species synthesis. Because genes associated with the anti-mycobacterial function of M1-MФs were enhanced in MDSCs following mab treatment, we propose that LILRB2 antagonism reprograms MDSCs from an immunosuppressive state towards a pro-inflammatory phenotype that kills Mtb. LILRB2 is therefore a novel therapeutic target for eradicating Mtb in MDSCs.

**Graphical Abstract:**
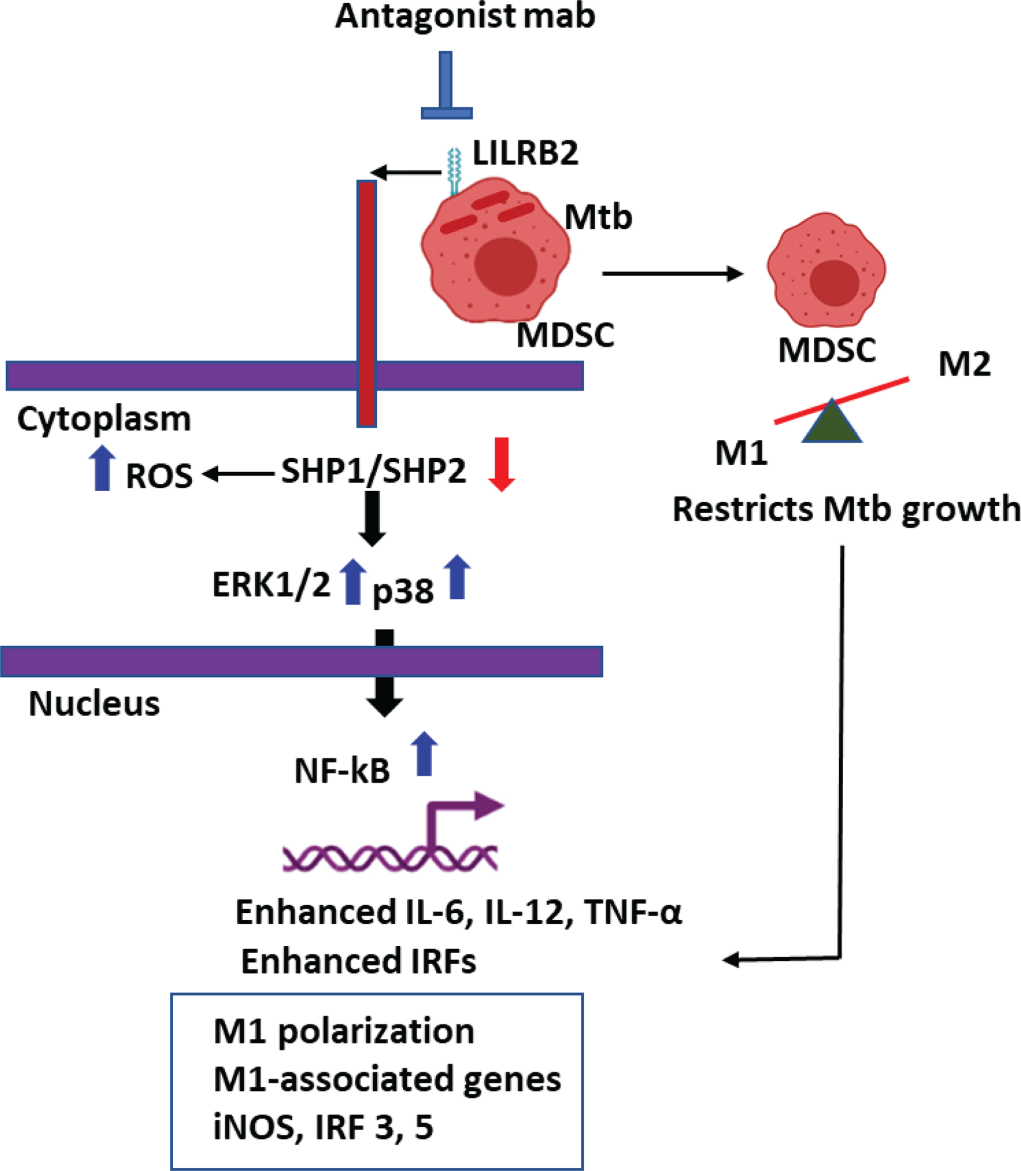
LILRB2 antagonism enhances killing of Mycobacterium tuberculosis in Myeloid derived Suppressor Cells (MDSCs).

## Introduction

*Mycobacterium tuberculosis* (Mtb) that causes tuberculosis (TB) kills about 1 million people each year. Macrophages (MФs) are the major niche for either replication or persistence of Mtb and require IFN-γ-mediated activation to kill Mtb through nitric oxide (NO), reactive oxygen species (ROS), and autophagy or phagosome–lysosome (PL) fusion mechanisms. MФs are heterogeneous in nature with M1-MФ and M2-MФ major subsets (1). We recently showed that Mtb infection induced striking transcriptomic changes in both rested and IFN-γ differentiated M1-MФs; the latter killed Mtb using NO and autophagy-dependent mechanisms. In contrast, IL-4-programmed M2-MФs allowed the growth of Mtb due to defects in these antimycobacterial mechanisms (2).

Emerging evidence suggests a critical role of myeloid-derived suppressor cells (MDSCs) in promoting immunosuppression in cancers, and they are a major target for immunotherapy of cancer (3). Curiously, MDSCs also arise during the pathogenesis of tuberculosis; they are found in the blood and lung granulomas of TB patients and thought to exacerbate disease severity by secreting anti-inflammatory cytokines besides a direct suppressive interaction with other immune cells (4–8). Although chronic mycobacterial infection triggers an expansion of MDSCs, less is known about MDSCs as an Mtb reservoir and strategies to eliminate the pathogen (9, 10). Whereas anti-TB immunity is regulated by the Th1/TH17/IFN-γ-dependent axis, during early and late stages of TB, a variety of suppressor cells including MDSCs and T and B regulatory cells (Tregs and Bregs) may emerge and aggravate the disease severity (9). We therefore sought to determine whether the suppressive function of MDSCs can be abrogated. We noted that MDSCs are either derived from monocytic (M-MDSCs) or neutrophilic (N-MDSCs) lineage (11, 12). The molecular mechanisms through which these phenotypes occur and their immune regulation during tuberculosis remain unclear (5, 13).

We recently described that the human leukocyte immunoglobulin-like receptors (LILRBs) and its mouse counterpart, paired immunoglobulin-like receptor B (PIR-B), are critical cell surface receptors that regulate the functional phenotype of MDSCs and MФs (14, 15). PIR-B is a key regulator for maintaining the M2 phenotype of tumor-infiltrating MDSCs, and we demonstrated for the first time that blockade of LILRB2 induces differentiation MDSCs into the M1 type of macrophages, altering the tumor microenvironment towards anticancer responses (16). In this direction, we developed agonist and antagonist antibodies against human LILRB2, which have the potential to enter clinical applications. Because TB requires prolonged chemotherapy and MDR-TB poses a challenge to treat, we hypothesized that LILRB2 modulation can be used to target Mtb containing MDSCs for controlling TB. Herein, we demonstrate that LILRB2-specific mab activation of MDSCs skews them into an M1-phenotype enhancing their ability to kill Mtb.

## Materials and Methods

### Statistics

Graphpad Prism was used to analyze data and obtain p-values. All experiments contained biological triplicates and were done at least two independent times.

### Study Approval

The procedures of sample collection and analyses were approved by HSC-SPH-12-0037.

### Blood Samples

Samples from healthy donors and TB patients were obtained following approved IRB procedures. Within TB patient and healthy controls, the samples were randomized for age, sex, and other demographics for unbiased analysis (**Supplementary Table 1**). From 10 ml of blood, 1 ml was subjected to RBC lysis, centrifuged at 400g for 5 min. PBS washed cells were stained with 100 µl of viability dye for 5 min. Cells were then washed with PBS + 1% FBS and blocked with 50 µl of PBS + 1% FBS containing human Fc block for 10 min, followed by 50–100 µl of antibodies (**Supplementary Table 1**) against various immune cells using our CyTOF (cytometry by time of flight or mass cytometry; HMRI) core. Cells were mixed and incubated for 30 min, and washed four times with PBS + 1% FBS followed by overnight fixation. From the remaining 9 ml of blood, PBMCs were also isolated using Ficoll®Paque Plus (GE Healthcare #17-1440-02) and were stained with antibodies as above and used for CyTOF analysis.

### Isolation of MDSCs From Peripheral Blood Monocytes

MDSCs were isolated as described previously (17). Briefly, PBMCs from the buffy coat of healthy donors were isolated using Ficoll®Paque Plus (GE Healthcare #17-1440-02), which resulted in a yield of about 200 million PBMCs. PBMCs were then subjected to CD14+ cell fractionation using an EasySep Human CD14+ positive selection kit (Stem cells; #17858) as per the manufacturer’s instructions. PBMCs from one buffy coat unit yielded about 80 million CD14+ cells. The CD14+ cells were cultured for 6 days in IMDM medium supplemented with 10% fetal bovine serum in the presence of GM-CSF (10 ng/ml) in combination with IL-6 (10 ng/ml) to induce their proliferation into Monocytic-MDSCs. M-MDSCs were then purified from CD14+ cells using the EasySep Human CD33 positive selection kit (Stem cells; #17876) as per the manufacturer’s instructions. The purity of M-MDSCs (CD15-CD14+CD33+HLADR-) was found to be >90% by flow cytometry (**Supplementary Figure 1**). The total yield for M-MDSCs was in the range of 16–20 million cells per donor from one unit of buffy coat. To test whether MDSCs naturally occurring in blood show bactericidal function after mab activation, for some experiments (**Figure 4**), CD33+ MDSCs were purified using FACsorting of PBMCs from healthy donors.

### Flow Cytometry

Flow cytometric analysis was done to assess the purity of M-MDSCs (henceforth referred to as MDSCs). Cells were stained with Fluorochrome-conjugated antibodies, FITC anti-human CD14 (Biolegend, #367115), PE anti-human CD15 (Biolegend, #301905), PE-Cy7 anti-human HLADR (Biolegend, #307615), APC anti-human CD33 (Biolegend, #366605), and Fixable Viability Stain 510 (BD Biosciences, #564406). Cells were analyzed on the Fortessa flow cytometer 202 (Beckton Dickinson), and the data were processed using FlowJo v10 software (Tree Star, Inc.).

### Infection and Colony-Forming Unit Counts of *M. tuberculosis* in MDSCs

MDSCs were plated at 10^6^ per well in 24-well adherent plates. Mtb H37Rv (ATCC 27294) was grown in Middlebrook 7H9 broth supplemented with 0.05% (v/v) Tween 80 and Middlebrook AODC enrichment (Difco, Becton Dickinson) to mid-log phase (OD 600 nm 0.6± 0.8). Early log phase cultures of Mtb suspensions were sonicated at 4 W for 60 s using a sonicator to prepare a uniform single-cell suspension and then MDSCs were infected with Mtb at a MOI of 1. After 4 h of infection, cells were washed and added with LILRB-2 mab (mab-B2) or its IgG control antibody for 18 h at 1 µg/ml per 10*6 MDSCs for 7 days. Mtb counts were expressed as log10 CFU counts averaged for triplicate wells of MDSCs per group or combination and experiments were repeated two times. p-value for differences in CFUs was determined using 1-way ANOVA using Tukey’s posttest.

### Effect of siRNA Blockade of Nitric Oxide and Autophagy on The Growth of Mtb in MDSCs

MDSCs were subjected to siRNA knockdown for autophagy using manufacturer’s instructions (18). The kits for human siRNAs (mixture of duplexes) were purchased from Origene technologies (Beclin-1 siRNA including scrambled controls: SR322490; iNOS siRNA: SR303202). MDSCs were treated with siRNA and the scrambled control, washed, rested, and added with Mtb (H37Rv) for 4 h of infection (MOI of 1). Cells were washed and added with mab-LILRB2/3 (1 µg/ml each) or isotype (1 µg/ml) and incubated as required. Mab was combined with inhibitors (1 µg/ml each) as needed: SHP1 inhibitor (IACS-13909; #HY-137092/CS-0136474) and SHP2 inhibitor (TPI-1 #HY-100463/CS-6450) were purchased from MCE Med Chem Express. At time points, cells were lysed in 0.05% SDS-PBS buffer and 10-fold dilutions were plated on 7H11 agar plates for CFU counts, which were read after 21 days of incubation. To assess the effects of NO or ROS on Mtb growth in MDSCs, replicate cultures of mab-treated or untreated MDSCs were incubated with diamino-fluorescein diacetate (DAN) fluorescent probe (1 µM) followed by fluorometry using Ascent Fluoroscan and relative light units (RLUs) plotted for triplicate wells per treatment group (19). Whole-cell ROS was similarly detected using fluorometry and dihydro-dichloro-fluorescein diacetate (1 µM) using live cells as described (20).

### Immunofluorescence Colocalization Assay for LC3 Labeling

Naive MDSCs were infected using gfp-expressing Mtb (MOI of 1) for 4 h, washed and treated using either mab-B2 or isotype, and incubated at 18 h. Post infection, the cells were washed, plated onto coverslips, fixed in 3.7% paraformaldehyde, and permeabilized using Tween 80-digitonin-BSA buffer as described before (18). A specific mab to LC3 (Cell Signaling #3868) was used to stain gfpMtb phagosomes followed by Alexa fluor 555 conjugates. Confocal images were acquired using an N90 Nikon microscope equipped with a Metaview RT deconvolution software. Colocalization was determined by scoring gfpMtb phagosomes of 25 MDSCs in triplicate chambers per treatment group (18, 21).

### qPCR Analysis of CD80, CD206, GBP2, IRF3, IRF5, and IRF7

Total RNA was extracted from control or treated MDSCs using the RNAeasy mini kit (Qiagen, Germany). RNA concentration and purity ratios (OD260/280, OD260/230) were measured using the NanoDrop ND-1000 spectrophotometer (Thermo Fisher Scientific, USA). cDNA synthesis was performed on a CFX96 Real-Time PCR System (Biorad, USA) using the 2X OneStep qRT-PCR Mastermix Kit (Applied Biosystems, USA) according to the manufacturer’s instruction. Quantitative PCR (qPCR) was performed using SYBR green probe and gene specific primers [CD80-5’->3’: CTCTTGGTGCTGGCTGGTCTTT Forward primer; 5’->3’: GCCAGTAGATGCGAGTTTGTGC Reverse primer; CD206-5’->3’: AGCCAACACCAGCTCCTCAAGA Forward primer; 5’->3’: CAAAACGCTCGCGCATTGTCCA Reverse primer; GBP2-5’->3’: AGCCAACACCAGCTCCTCAAGA Forward primer; 5’->3’: CAAAACGCTCGCGCATTGTCCA Reverse primer; IRF3-5’->3’: TCGTGATGGTCAAGGTTGT Forward primer; 5’->3’: AGGTCCACAGTATTCTCCAG Reverse primer; IRF5-5’->3’: ATGCTGCCTCTGACCGA Forward primer; 5’->3’: GCCGAAGAGTTCCACCTG Reverse primer; IRF7-5’->3’: GAGCCCTTACCTCCCCTGTTAT Forward primer; 5’->3’: CCACTGCAGCCCCTCATAG Reverse primer]. Threshold cycle numbers were transformed to ΔΔCt values, and the results were expressed relative to the reference gene, β-actin, and GAPDH. Gene expression data were performed using GraphPad Prism ver. 6.0 suite (GraphPad Software). Student’s t-test was used for means comparison between both uninfected and Mtb-infected MDSCs. Significance was set at the 0.05 level.

### Wes-Simple Capillary Electrophoresis

MDSCs were collected and centrifuged for 5 min at 400g and lysed by using RIPA buffer in the presence of protease inhibitors. For the analysis of proteins, the quantitative Wes capillary immunoassay was used, in which the lysates were separated and detected using Wes separation capillary cartridge 12–230 kDa along with the Wes Anti-Rabbit Detection Module (Simple Western system and Compass Software, Protein Simple). (**Supplementary Figure 2**) In brief, glass microcapillaries were loaded with stacking and separation matrices followed by sample loading. During capillary electrophoresis, proteins were separated by size and then immobilized to the capillary wall. Samples were loaded at 1 mg/ml dilution and the primary rabbit antibody was used at a dilution of 1:50 and β-actin (Rabbit monoclonal, Sigma-Aldrich # SAB5600204) was used at 1:50 dilution. The antibodies for iNOS (Abclonal, #A0312) and STAT1 (#14994T), p-STAT1 (#9167S), ERK1/2 (#4695T), p-ERK1/2 (#4370 T), p38 MAPK (# 8690T), p-p38 MAPK (#4511T), NF-kB (#8242T), and p-NF-kB (#3033T) were purchased from Cell Signaling Technology. Data were analyzed with the Compass software (version 2.6.7). The area under the curve (AUC), which represents the signal intensity of the chemiluminescent reaction, was analyzed for all the antibodies and β-actin. Values given for protein expression were normalized to β-actin. Quantitation of protein levels (area under each peak; arbitrary units [A.U.]) were performed using the Compass software (version 2.6.7).

### Western Blot analysis

For the analysis of LC3B protein, a traditional Western blot was used as the protein was of small molecular weight. Protein concentrations were determined using the Bradford Assay Reagent (Thermo Scientific: cat. no. 23238) and 25 µg of total protein was loaded per well of 4%–20% Mini-Protean TGX gels (Bio-Rad #4561095) and transferred to nitrocellulose membranes (Trans-Blot Turbo Transfer pack, Bio-Rad #1704158) using the Bio-Rad trans-blot turbo transfer system. Antibody against LC3B was used at a dilution of 1:500 (LC3B Antibody; Novus Biologicals: #NB100-2220SS). Secondary antibody was added (1:1,000 Anti-rabbit IgG, HRP-linked Antibody, Cell Signaling #7074s), and the membranes were then developed using an ECL kit. Densitometry analysis was done using ImageJ software and LC3 band density was normalized relative to GAPDH/β-actin (Anti-GAPDH antibody, Rabbit monoclonal, Sigma-Aldrich #SAB5600208) or β-actin (Anti-β Actin antibody, Rabbit monoclonal, Sigma-Aldrich #SAB5600204).

## Results

### Distribution of Monocytic and Neutrophilic Myeloid-Derived Suppressor Cells in PBMCs and Whole Blood of Tuberculosis Patients and Healthy Donors

**Figure 1:**
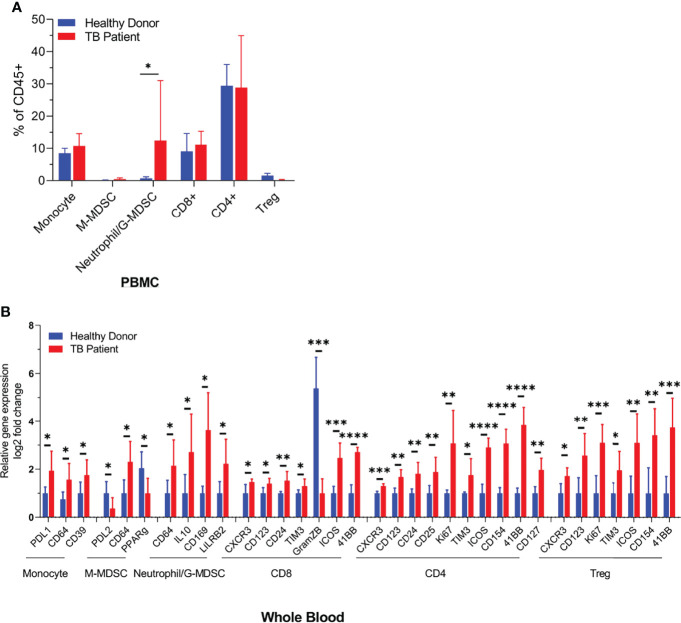
Distribution of monocyte and neutrophil-derived myeloid-derived suppressor cells (MDSCs) in the blood of healthy donors and tuberculosis patients. Blood samples from either healthy donors (*n* = 5) or TB patients (*n* = 5) were analyzed using CyTOF (mass cytometry) for immune cell populations. N-MDSCs are increased in TB patients who also show a reduced activation of CD8 T cells (*p < 0.05, **p < 0.005, ***p < 0.0005, ****p < 0.01, two-tailed *t*-test). Individual CyTOF profiles, biomarkers, and sample data are shown in **Supplementary Table 1** and **Supplementary Figure 3**.

During natural infection, Mtb are inhaled and phagocytized by alveolar MФs. They infiltrate into the lung parenchyma and secrete chemokines to recruit interstitial and blood-deirved monocyte-MФs, neutrophils, dendritic cells, and various subsets of T cells to form an organzied structure called granuloma (22). TB granulomas contain a central core of Mtb-infected macrophages surrounded by macrophages, T cells, DCs, and PMNs. Interestingly, MDSCs also occur in and around tuberculosis granulomas and in the peripheral blood of TB patients (10). To determine their frequency in circulation, both PBMCs purified on Ficoll and whole blood RBC lysis preparations, from tuberculosis patients (n = 5) and heathy donors (n = 5), were analyzed using CyTOF. **Figure 1** illustrates that neutrophilic/granulocytic-type N-MDSCs were higher among PBMCs of TB patients and there was a marked reduction in the activation of CD8+T cells compared to CD4 T cells. Although M-MDSCs were lesser in PBMCs, herein, we pursued their analysis of because of their potential role during TB. CyTOF analysis was done to confirm the presence of N-MDSCs and M-MDSCs in samples of TB patients and compared with donors; **Supplementary Figure 3** shows the t-SNE plots of immune cell composition and **Supplementary Table 1** indicates the clinical status of TB patients and controls.

### LILRB2-Specific Mab-Mediated Antagonism of MDSCs Enhances Their Bactericidal Function Through Nitric Oxide and Reactive Oxygen Species

**Figure 2:**
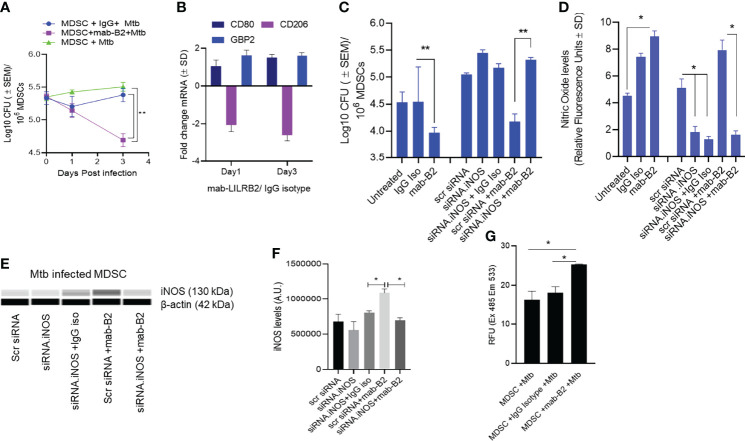
A monoclonal antibody to leukocyte immunoglobulin-like receptor B2 (LILRB2) enhances the killing of *Mycobacterium tuberculosis* (Mtb) in MDSCs through nitric oxide. **(A)** CD14^+^/CD33^+^ bead-purified M-MDSCs derived from GM-CSF/IL-6 cultured PBMCs of a healthy donor were infected for 4 h with Mtb H37Rv (MOI = 1), washed, and activated using either a monoclonal antibody to LILRB2 (mab-B2; 1 µg/ml) or its IgG isotype (1 µg/ml) or left untreated. MDSC lysates were plated at indicated time points for Mtb CFUs on 7H11 agar (triplicate wells per point; **p < 0.007 1-way ANOVA and Tukey’s posttest; 1 of 2 similar experiments shown). **(B)** At indicated time points, MDSC cell lysates in trizol were collected for qPCR for CD80, CD206, and Guanylate binding protein-2 biomarkers (1 of 2 similar experiments shown). **(C)** MDSCs were treated with siRNA vs. inducible nitric oxide synthase (*iNOS*) or its scrambled control followed by 4 h Mtb infection (MOI = 1), washed, and activated using mab-B2 or its isotype and incubation for 3 days before CFU counts (triplicate wells per point; **p < 0.009 1-way ANOVA; 1 of 2 similar experiments shown). **(D)** Replicates of panel C were incubated with Diaminofluorescein diacetate followed by a fluorometric detection of soluble nitrite in medium (triplicate wells per point; *p < 0.009 *t*-test; 1 of 2 experiments shown). **(E)** Cell lysates of panel C were tested for iNOS protein using ProteinSimple-Wes capillary electrophoresis. Expression of iNOS was normalized to β-actin. **Supplementary Figure 2**illustrates the Wes-capillary gel analysis. **(F)** Densitometry of proteins for panel E (*p < 0.001 *t*-test). **(G)** Relative production of reactive oxygen species (ROS) by MDSC with and without treatment of mab-B2 following Mtb infection. ROS was detected using dihydro-dichloro-fluorescein aceate and fluorometry (*p < 0.001 *t*-test).

Using CD14 and CD33 bead fractionation, M-MDSCs were purified from PBMCs of healthy donors. They were infected for 4 h with Mtb, washed, and then treated with either mab-B2 or its isotype followed by incubation for Mtb growth assay. **Figure 2A** indicates that the Mtb CFU counts progressively declined over 3 days, whereas their numbers remained stable in untreated or IgG isotype-treated MDSCs. Because MDSCs were infected for 4 h, we sought to determine if there are differences in the uptake of Mtb after a 4-h infection. Microscopic counting of Mtb within MDSCs using acid fast mycobacterial stains did not show a significant difference in the uptake by naive MDSCs (day 0 CFU; **Figure 2A**). Thus, we demonstrate that LILRB2 mab-mediated antagonism rapidly enhances the killing of Mtb, increasing to a 0.8-log10 decline in CFU by 3 days.

Earlier, we have shown that mab-B2 blockade of LILRB2 switches the MDSC phenotype into an M1-MФ phenotype (16). To determine whether mab-B2 induces a similar switch, Mtb-infected MDSCs were collected on days 1 and 3 post mab-B2 treatment, and tested for the mRNA expression of CD80 and CD206, which are markers of M1-MФs and M2-MФs, respectively (23). In addition, we evaluated mRNA for Guanylate binding protein 2 (GBP2), which is an IFN-γ-inducible marker associated with the control of mycobacterial infection in mice (24–26). Mtb-infected MDSCs treated with mab-B2 showed an upregulation of CD80 and a downregulation of CD206 consistent with an M1-MФ phenotype (**Figure 2B**). GBP2, a biomarker for M1-MФs, was maintained between day 1 and day 3 after LILRB2 blockade.

Since MФs can kill Mtb using NO, we sought to determine whether mab-B2 induced iNOS, which, in turn, can generate NO to boost the bactericial funciton of MDSCs. iNOS synthesis in MDSCs was therefore blocked using siRNA vs. iNOS followed by Mtb infection and incubation with mab-B2 or IgG isotype. Treatment with mab-B2 reduced the Mtb viable counts compared to IgG-treated MDSCs (**Figure 2C**). Moroever, mab-B2-induced intracellular killing of Mtb was abrogated when iNOS was inhibited (**Figures 2C, D**). Replicate cultures of similarly treated MDSCs showed that mab-B2-enhanced NO was confirmed by the presence of nitrite in the medium detected using a diaminonapthalene (DAN) fluorescent indicator and fluorometry (19) (**Figure 2D**). Western blot analysis of iNOS confirmed that the barely detetable iNOS protein in Mtb-infected MDSCs was enhanced after mab-B2 treatment (**Figures 2E, F**). Specific siRNA knockdown of iNOS but not scramled siRNA reduced the levels of iNOS protein (**Figure 2F**). We note here that LILRB2 signals through the src-homolgy containing phosphatases (SHP)-1 and 2 (14). Because SHP-1 mediates negative reguation of iNOS (27), we suggest that mab-B2-LILRB2 blockade suppressed SHP-1 to increase NO, essentially switching MDSCs to an M1-MФ-like phenotype and controlling Mtb growth.

Since MФs can also induce the killing of Mtb using ROS, we sought to determine whether blockdade of LILRB2 induces the ROS; ROS was marginally increased in Mtb-infected MDSC after mab-B2 treatment (**Figure 2G**). Together, these data confirm that mab-B2 induces Mtb-infected MDSCs to switch into an M1-MФ like phenotype, increasing NO and ROS and controlling the growth of Mtb.

### LILRB2 Antagonism-Induced Bactericidal Function of MDSCs Is Independent of Autophagy

**Figure 3:**
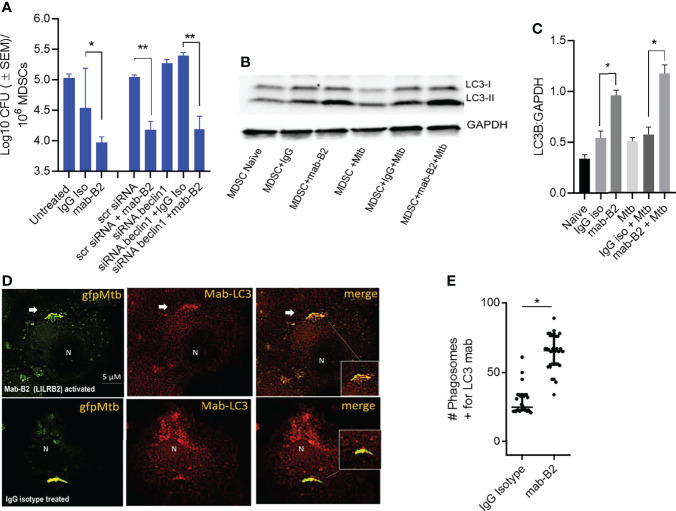
LILRB2 antagonism effects on the bactericidal function of the MDSCs are independent of autophagy. **(A)** CD14^+^/CD33^+^ bead-purified M-MDSCs derived from GM-CSF/IL-6 cultured PBMCs from donors were subjected to autophagy blockade using siRNA vs. beclin1 (*ATG6*) or its scrambled control, followed by infection with Mtb H37Rv (MOI = 1), washed, and incubated after activation with mab-B2 or isotype. MDSC lysates were plated on day 3 for Mtb CFUs on 7H11 agar (triplicate wells per point; *p* < 0.008, 1-way ANOVA and Tukey’s posttest; 1 of 2 similar experiments shown). **(B)** Lysates of cells from panel A were collected at 18 h and analyzed using Western blot for the autophagy marker, microtublue-associated light chain-3 (LC3). Lipidation is indicated by LC3-II. **(C)** Densitometry of cells from panel **B** indicates the expression of LC3 normalized to GAPDH. (*p < 0.01 *t*-test). **(D)** M-MDSCs were infected with *gfp*Mtb (4 h MOI = 1) followed by fixation in 3.7% paraformadehyde and staining using mab to LC3 followed by Alexafluor590 anti-IgG conjugate. Images were acquired using an N90 Nikon scope with Metaview deconvolution software. **(E)** Data show quantitation of *gfp*Mtb phagosomes colocalizing with mab-LC3 staining (*p < 0.05, **p < 0.005, *t*-test; 1 of 2 similar experiments shown).

Besides oxidants like NO and ROS, MФs can also kill Mtb by sorting Mtb phagosomes to lysosomes through either Rab GTPase and SNARE-dependent phagosome–lysosome (PL) fusion or through autophago-lysosome fusion regulated by *ATGs* (28, 29). To determine whether autophagy plays a role after LILRB2 antagonism, MDSCs were subjected to siRNA knockdown of beclin1 (*ATG6*) and scrambled siRNA controls. Following Mtb infection, MDSCs were treated with mab-B2 or IgG isotype and incubation for Mtb growth assay. Unexpectedly, siRNA-mediated autophagy blockade of MDSCs did not reverse the Mb growth in mab-B2-treated cells (**Figure 3A**). This was interesting because mab-B2 induced an enrichment of the autophagy marker protein, microtubule-associated light chain-3 (LC3-II) (**Figures 3B, C**). During autophagolysosome fusion, the lipidated form of LC3-II is enriched on the phagosomes of Mtb. Fluorescent immunostaining using antibodies to LC3 also confirmed that many gfpMtb phagosomes were enriched for LC3 labeling (**Figures 3D, E**). Therefore, a lack of autophagy-mediated killing of Mtb in MDSCs was in contrast with LC3 enrichment of their phagosomes. However, we note that Mtb phagosomes can transiently label with LC3 even without causing the autophagic flux, which delivers them to lysosomes (30). Alternatively, Mtb may evade mab-B2-induced autophagy through secretion of sapM phosphatase, which cleaves phosphatidyl-3 inositol phosphate (PI3P) that is associated with phagosomes preventing autophagolysosome fusion. In this direction, we reported that a Δ*fbpA*Δ*sapM* mutant of Mtb is lysosome fusion competent (31, 32).

### LILRB2 Antagonism in MDSCs Renders Them Bactericidal for a Subsequent Mtb Infection

**Figure 4:**
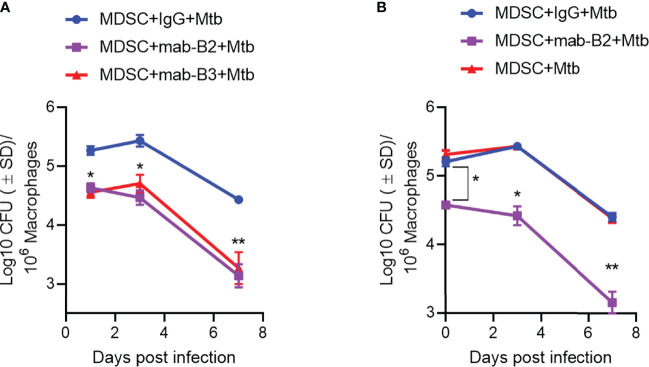
Prior treatment of MDSCs with monoclonal antibodies to leukocyte immunoglobulin-like receptors B2 and B3 (LILRB2/3) enhances their bactericidal function against *Mtb.* ***(*A, B*)*** CD33^+^ flow sorted MDSCs from donor PBMCs **(A)** or CD14^+^/CD33^+^ bead-purified M-MDSCs derived from GM-CSF/IL-6 cultured PBMCs from donors **(B)** were activated or not with monoclonal antagonist antibodies to LILRB2 (mab-B2) or LILRB3 (mab-B3) followed by infection with Mtb H37Rv (MOI = 1) and incubation over time. Lysates were plated at indicated time points for Mtb CFUs on 7H11 agar (*, **p < 0.007 1-way ANOVA and Tukey’s posttest; 1 of 2 similar experiments shown).

Because LILRB2 blockade led to increased killing of Mtb by MDSCs (**Figure 2A**), we sought to determine whether it can also “pre-condition” MDSCs to prevent a subsequent Mtb infection and growth. First, CD33+ MDSCs were purified using FACsort from donor PBMCs and treated with mab-B2 and LILRB3-specific mab-B3, followed 1 day later by infection with Mtb and growth assay over 7 days. **Figure 4A** indicates that mab-B2 and mab-B3 (antagonistic for LILRB3) treatment signficantly reduced the uptake of Mtb by MDSCs on day 1. However, both mabs still activated MDSCs to kill Mtb over 7 days. Next, CD14+CD33+ MDSCs bead purified from PBMCs cultured in GM-CSF/IL-6 (used for **Figures 1**–**4**) were treated using mab-B2/3 followed by Mtb infection and CFU assay. Similar to MDSCs directly purified from blood (**Figure 4A**), culture-grown MDSCs also enhanced the killing Mtb upon LILRB2/3 blockade despite the difference in uptake of Mtb on day 1 (**Figure 4B**).

### LILRB2 Antagonism Activates ERK1/2, p38 MAPK, and NF-κB in Mtb-Infected MDSCs Increasing Inflammatory Cytokines

**Figure 5:**
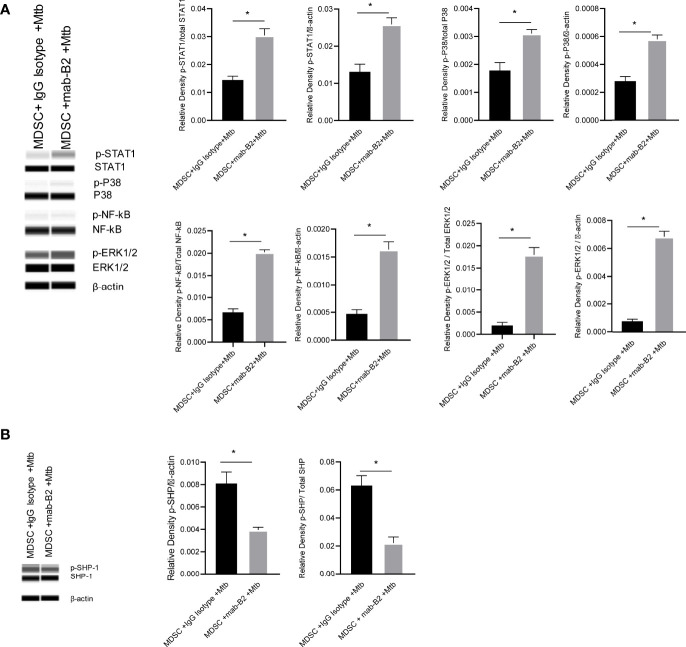
LILRB2 antagonism drives the STAT1/NF-κB inflammatory pathway in Mtb-infected MDSCs. CD14^+^/CD33^+^ bead-purified M-MDSCs derived from GM-CSF/IL-6 cultured PBMCs from donors were infected for 4 h with Mtb H37Rv (MOI = 1), washed, and activated using either a monoclonal antibody to LILRB2 (mab-B2) or its IgG isotype. MDSC lysates were collected at 18 h post infection in RIPA buffer for Western blots and densitometry. **(A)** Immunoblot of phosphorylated STAT1, p38 MAPK, NF-κB, and ERK1/2 in MDSCs treated as above. Densitometry shown to the right (*p < 0.02, unpaired *t*-test). **(B)** Immunoblot of phosphorylated SHP1 in MDSCs treated as above (*p < 0.02 unpaired *t*-test) (SHP2 was not affected; not shown; 1 of 2 similar experiments shown).

Inflammatory phenotypes of M1-MФs are associated with NF-κB/STAT1 activation (33). Because LILRB2 antagonism led to an M1-type switch in MDSCs (**Figure 2B**), we evaluated downstream signaling mechanisms after mab treatment in Mtb-infected MDSCs. LILRB2 mab-treated MDSCs showed increased ERK1/2, p38 MAPK, and NF-κB/STAT1 phosphorylation in Mtb-infected MDSCs after Mtb infection compared to IgG controls (**Figure 5A**). We reported earlier that LILRB receptors constitutively recruit and activate SHP-1 (SH2 domain-containing protein tyrosine phosphatase-2) in macrophages (16). Consistent with this observation, LILRB2-associated SHP-1 phosphorylation was reduced in mab-B2 treated Mtb-infected MDSCs compared to IgG controls (**Figure 5B**). These data suggest that LILRB2 antagonism disrupts SHP-1 and increases downstream inflammatory signaling cascades through phosphorylated ERK1/2, p38 MAPK pathways, and NF-κB and STAT1 transcription factors.

### LILRB2 Antagonism and SHP1/2 Phosphatase Inhibition Synergize to Kill Mtb in MDSCs

**Figure 6:**
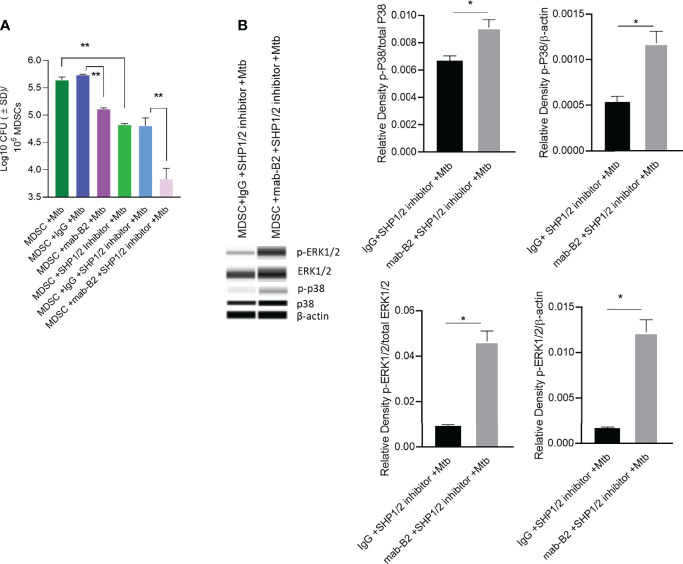
Mab-B2 antagonism of LILRB2 and pharmacological inhibition of SHP1/2 synergistically boost killing of Mtb in MDSCs. CD14^+^/CD33^+^ bead-purified M-MDSCs derived from GM-CSF/IL-6 cultured PBMCs from donors were infected for 4 h with Mtb H37Rv (MOI = 1), washed, and activated using either a monoclonal antibody to LILRB2 (mab-B2) or its IgG isotype with or without added SHP-1 and SHP-2 inhibitors (1 µg/ml each per 10*6 cells). **(A)** MDSC lysates collected 18 h after infection were plated for Mtb CFUs on 7H11 agar (**p < 0.002 by 1- way ANOVA with Tukey’s posttest; 1 of 2 similar experiments shown).**(B)** Lysates collected from replicate cultures of MDSCs were analyzed using Wes simple Western blot for phosphorylated ERK1/2 and p-38 kinases, and densitometry is shown to the right (*p < 0.02, unpaired *t*-test).

Because LILRB2 antagonism in Mtb-infected MDSCs reduced SHP-1 phosphorylation (**Figure 5B**), we pharmacologically blocked SHP1/2 to boost mab-B2-mediated antagonism. To demonstrate synergy, Mtb-infected MDSCs were plated for CFUs by 18 h post treatment. Remarkably, mab-B2 in combination with TPI-1 and IACS, which are inhibitors of SHP-1 and SHP-2, respectively, showed a rapid and synergistic reduction of Mtb burden in MDSCs. Synergy suggests that LILRB2 may not solely go through the SHP1/2 pathway; alternatively, other LILRB members may signal through SHP1/2. MDSCs treated with mab-B2 showed 0.6 log10 reduction of Mtb CFU compared to IgG control by day 3, whereas we found ~1.8 log reduction of Mtb CFUs in the mab-B2–SHP1/2 inhibitor combination (**Figure 6A**). This observation correlated with an increased phosphorylation of ERK1/2 and p38 MAPK activation compared to MDSC treated with IgG and SHP-1 and SHP-2 inhibitors (**Figure 6B**). Thus, LILRB2 antagonism through mab in combination with the disruption of SHP1/2 enhances ERK1/2, p38 MAPK, and the robust killing of intracellular Mtb.

### LILRB2 Antagonism Enhances IRF Transcription Factors and Pro-Inflammatory Cytokine Secretion by Mtb-Infected MDSCs

**Figure 7:**
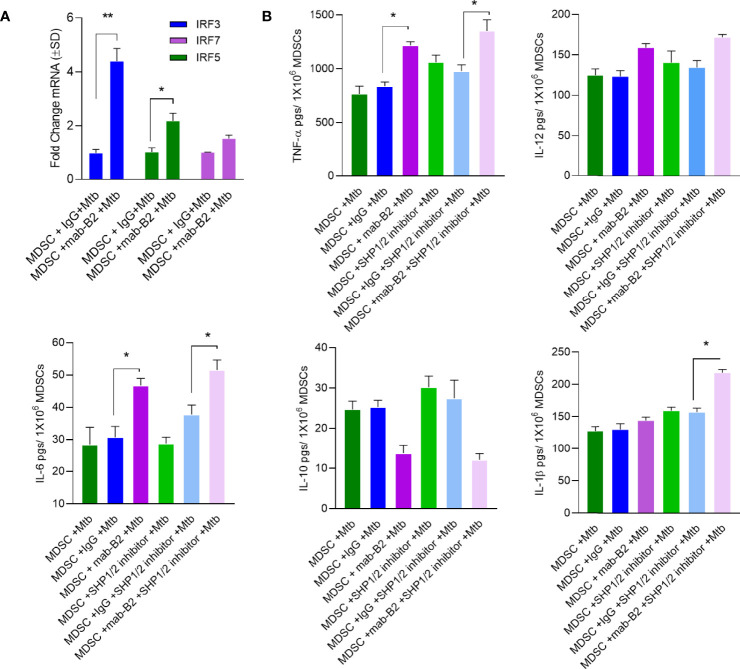
LILRB2 antagonism enhances inflammatory cytokines in Mtb-infected MDSCs. CD14^+^/CD33^+^ bead-purified M-MDSCs derived from GM-CSF/IL-6 cultured PBMCs from donors were infected for 4 h with Mtb H37Rv (MOI = 1), washed, and activated using either a monoclonal antibody to LILRB2 (mab-B2; 1 µg/ml/10*6 cells) or its IgG isotype (1 µg/ml/10*6 cells) and SHP 1/2 inhibitors (1 µg/ml of each per 10*6 cells). Cells were lysed in trizol for qPCR and supernatants were tested for cytokines. **(A)** qPCR mRNA quantitation after mab-B2 or IgG treatment of Mtb-infected MDSCs at 18 h post infection (*p < 0.02 **, unpaired *t*-test). **(B)** Supernantants were collected at 18 h post infection and cytokines were assayed using sandwich ELISA (*p < 0.01 **, unpaired *t*-test).

A downstream mechanism of SHP2 is its inhibition of transcriptional activity of IRF-1 and IRF-8 at the IFN-β promoter. Whereas IRF1, IRF5, and IRF8 promote M1-type polarization of macrophages, IRF3 and IRF4 promote M2 differentiation (34). LILRB2 treatment with mab-B2 upregulated IRF3 and IRF5 compared to IgG controls in Mtb-infected MDSCs (**Figure 7A**), confirming a switch of MDSCs into the M1 phenotype. To determine whether IRF changes are associated with cytokine secretion, MDSCs were treated with mab-B2, SHP1/2 inhibitors (TPI-1 and IACS), or their combination followed by cytokine assay. Treatment with mab-B2 alone was enough to increase pro-inflammatory TNF-α, whereas a combination of mab-B2 blockade and SHP1/2 inhibitor led to elevated TNF-α, IL-6, and IL-1β (**Figure 7B**). In contrast, mab-B2 or SHP1/2 inhibitors decreased the levels of anti-inflammatory IL-10. We recall here that both IL-1β and TNF-α are macrophage-activating cytokines (35, 36).

## Discussion

MDSCs are a distinct class of suppressor cells that mediate immune regulation during chronic inflammation and infectious diseases (9, 10). In the context of tuberculosis, the lung granulomas seem to contain MDSCs, presumably of both monocytic and granulocytic origin (7) (10). In vitro cultures of MDSCs allow increased growth of Mtb, even though they seem to secrete more inflammatory cytokines (37). Because MDSC-secreted cytokines can affect the function of both MФs and T cells within the micro-environment of granulomas, they can be targets for immunotherapy (38). In this study, we have investigated a novel LILRB2/3 receptor-dependent pathway in MDSCs to enhance their ability to kill Mtb and prevent exacerbation of TB.

MDSCs express myeloid activating and inhibitory receptors, including the LILR-A/B family (14). The paired immunoglobulin-like receptor B (PIR-B) is the only mouse receptor ortholog of the human LILRB family. Previously, we demonstrated that reprogramming of the MDSCs using LILRB2 blockade led to increased anticancer responses and such cells expressed an M1-like gene expression (16). Since M1-MФs can reduce the Mtb growth better than M2-MФs (2), we hypothesized that LILRB2 blockade can induce an M1-like switch in MDSCs facilitating a better control of Mtb growth.

First, we confirmed that MDSCs do occur in patients with tuberculosis, although the PBMCs seemed to contain a higher number of G-MDSCs (**Figure 1**). We continued our studies using M-MDSCs because macrophages dominate in the TB granulomas of lungs, and their sequestration in granulomas may render them less detectable in peripheral blood. Using in vitro cultures of PBMC-derived M-MDSCs, we found that LILRB2 blockade led to an increased killing of Mtb (**Figure 2A**). Importantly, mab-B2-treated MDSCs upregulated CD80, a marker of M1-MФs, and downregulated the CD206 biomarker of M2-MФs (**Figure 2B**). Intriguingly, increased bactericidal function of LILRB2-antagonized MDSCs was dependent on NO, and such cells also had increased ROS (**Figure 3**). On the other hand, blockade of autophagy had no effect on the growth of Mtb in mab-B2-treated MDSCs, although Mtb phagosomes still showed an increase in LC3 labeling (**Figure 4**). We recall here that Mtb phagosomes can acquire a transient labeling of LC3, but can also actively evade autophagolysosomal fusion in macrophages (39).

MDSCs are known to produce elevated levels of NO through iNOS (40), and interestingly, LILRB2 mab treatment (**Figures 2C–F**) enhanced NO response, which supports the concept of mab therapy which can perhaps clear MDSCs of Mtb organisms. However, we note that increased expression of NO and IDO by MDSCs and other cells may also suppress the T cell-mediated adaptive immune responses during TB (41, 42). Therefore, mab therapy of MDSCs can enhance bactericidal function but may also interfere with the adaptive arm of T cell-mediated anti-TB immunity. It is also possible that the unwanted side effect of adaptive immune cell suppression may be beneficial depending on the stage of TB; mabs may reduce pathologic inflammation and prevent lung damage during chronic or acute TB. This concept needs to be investigated using animal models of TB. We recall here that LILRB2 is also expressed by many other innate cells, including mesenchymal stem cells, neutrophils, and MФs, all of which can harbor Mtb (43, 44). The curative effects of LILRB2/3 mabs on these cell populations need to be determined.

Of note, we tested LILRB2 blockade both before and after Mtb infection because patients with TB and MDR-TB can be targeted using mab-B2 immunotherapy, and in addition, people at risk of TB can perhaps be given mab-B2 injections. We found that prior blockade of LIRB2/3 using mabs led to a moderate decrease in the uptake of Mtb organisms, which is consistent with the report that LILRB3 may affect FcR-mediated phagocytosis in macrophages (45). This line of research requires additional inevstigation. We also noted that isotype IgG by itself caused a moderate decline in the CFU counts of Mtb when added prior to but not after Mtb infection (**Figure 2A** vs. **Figures 4A, B**). This is likely because, when mab is added first, it may suppress Fc-R-dependent uptake of Mtb, a known non-opsonic pathway for mycobacterial uptake (46, 47). Nonetheless, mab-B2 induced a robust deline in Mtb viability compared to IgG treatment (**Figures 4A, B**).

Our earlier studies show that LILRB2 blockade suppresses the SHP1/2 phosphorylation, inducing an M1 phenotype through enhanced ERK1/2 and p38 MAPK phosphorylation and downstream activation of NF-kB and STAT-1 (16). Consistent with these findings, mab-B2 antagonism of LILRB2 in Mtb-infected MDSCs also led to enhanced STAT1, p38 MAPK, NF-κB, and ERK1/2 phosphorylation (**Figure 5A**), correlating with reduced SHP1/2 phosphorylation (**Figure 5B**). The increased STAT1 phosphorylation after mab-B2 blockade in MDSCs assumes importance because, during progressive Mtb infection of mouse macrophages, the levels of unphosphorylated STAT1 increases facilitating pathogen survival (48). Likewise, virulent Mtb shows immune evasion reducing ERK1/2 (49) and p38 MAPK phosphorylation (50), both of which increase Mtb survival in macrophages. We conclude that mab-B2 exerts its therapeutic effect through an enhanced phosphorylation of pivotal kinases and transcription factors in MDSCs.

Finally, we noted that activation of SHP1/2 is a key event downstream of LILRB2 and hypothesized that a combination of antagonism through mab-B2 and pharmacological blockade of SHP1/2 may have a synergistic effect on MDSCs. Indeed, SHP inhibitors are known to boost anticancer agents affecting MDSCs (51). We found a strong synergy between mab-B2 and a combination of SHP1/2 inhibitors (**Figure 6A**) that was associated with enhanced phosphorylation of p38 MAPK and ERK1/2 (**Figure 6B**). These data suggest that mab-B2 and SHP1/2 blockade could pave the way for a more effective immunotherapy for drug-sensitive and MDR-Mtb organisms. Since MDSCs can affect bystander immune cells through cytokine secretion, we analyzed cytokine profiles after LILRB2 antagonism. A combination of mab-B2 and SHP1/2 blockade enhanced Th1 (TNF-α, IL-6, and IL-1β) cytokine secretion and reduced 1L-10 (**Figure 7**). Because these cytokines perform anti-tuberculosis function, we conclude that LILRB2 is an attractive MDSC-related therapeutic target for tuberculosis control.

Despite the intriguing therapeutic effects of mabs to LIRB2/3, we note the following caveats. The number of MDSCs infiltrating to lungs could be limited and other myeloid cells that may or may not express LILRB2 like mesenchymal stem cells, dendritic cells, neutrophils, and M2-MФs may also contribute to immunosuppressive activity during TB (52) (53). Whether LILRB2/3 are expressed by the latter and amenable for mab therapy remains to be elucidated using ex vivo models and mice. Nonetheless, we anticipate that LILRB2/3-dependent signaling mechanisms are attractive therapeutic targets for the control of TB.

## Data Availability Statement

The original contributions presented in the study are included in the article/**Supplementary Material**. Further inquiries can be directed to the corresponding authors.

## Ethics Statement

The procedures of sample collection and analyses were approved by HSC-SPH-12-0037, UTHSC School of Public Health, Brownsville, TX, USA. The patients/participants provided their written informed consent to participate in this study.

## Author Contributions

VS, AK, YX, SM, LZ, and AM conducted experiments and acquired data. BR provided reagents. P-YP and S-HC designed and analyzed data. CJ designed the experiments and wrote the manuscript. All authors contributed to the article and approved the submitted version.

## Conflict of Interest

The authors declare that the research was conducted in the absence of any commercial or financial relationships that could be construed as a potential conflict of interest.

## Publisher’s Note

All claims expressed in this article are solely those of the authors and do not necessarily represent those of their affiliated organizations, or those of the publisher, the editors and the reviewers. Any product that may be evaluated in this article, or claim that may be made by its manufacturer, is not guaranteed or endorsed by the publisher.

## References

[1] Mills CD. M1 and M2 Macrophages: Oracles of Health and Disease. Crit Rev Immunol (2012) 32:463–88. doi: 10.1615/CritRevImmunol.v32.i6.10 23428224

[2] Khan A Zhang K Singh VK Mishra A Kachroo P Bing T. Human M1 Macrophages Express Unique Innate Immune Response Genes After Mycobacterial Infection to Defend Against Tuberculosis. Commun Biol (Nature) (accepted) (2022) 5:40. doi: 10.2139/ssrn.3750667 PMC9119986 35590096

[3] Gabrilovich DI. Myeloid-Derived Suppressor Cells. Cancer Immunol Res (2017) 5:3–8. doi: 10.1158/2326-6066.CIR-16-0297 28052991 PMC5426480

[4] Tesi RJ. MDSC; the Most Important Cell You Have Never Heard of. Trends Pharmacol Sci (2019) 40:4–7. doi: 10.1016/j.tips.2018.10.008 30527590

[5] Magcwebeba T Dorhoi A du Plessis N. The Emerging Role of Myeloid-Derived Suppressor Cells in Tuberculosis. Front Immunol (2019) 10:917. doi: 10.3389/fimmu.2019.00917 31114578 PMC6502992

[6] Garg A. Analysis of Antimicrobial Activity of Monocytic Myeloid-Derived Suppressor Cells in Infection With Mycobacterium Tuberculosis and Human Immunodeficiency Virus. Methods Mol Biol (2021) 2236:115–27. doi: 10.1007/978-1-0716-1060-2_11 PMC7845914 33237545

[7] Obregon-Henao A Henao-Tamayo M Orme IM Ordway DJ. Gr1(int)CD11b+ Myeloid-Derived Suppressor Cells in Mycobacterium Tuberculosis Infection. PloS One (2013) 8:e80669. doi: 10.1007/978-1-0716-1060-2_11 24224058 PMC3815237

[8] du Plessis N Loebenberg L Kriel M von Groote-Bidlingmaier F Ribechini E Loxton AG. Increased Frequency of Myeloid-Derived Suppressor Cells During Active Tuberculosis and After Recent Mycobacterium Tuberculosis Infection Suppresses T-Cell Function. Am J Respir Crit Care Med (2013) 188:724–32. doi: 10.1164/rccm.201302-0249OC 23885784

[9] Dorhoi A Du Plessis N. Monocytic Myeloid-Derived Suppressor Cells in Chronic Infections. Front Immunol (2017) 8:1895. doi: 10.3389/fimmu.2017.01895 29354120 PMC5758551

[10] Dorhoi A Kaufmann SH. Versatile Myeloid Cell Subsets Contribute to Tuberculosis-Associated Inflammation. Eur J Immunol (2015) 45:2191–202. doi: 10.1002/eji.201545493 26140356

[11] Negorev D Beier UH Zhang T Quatromoni JG Bhojnagarwala P Albelda SM. Human Neutrophils can Mimic Myeloid-Derived Suppressor Cells (PMN-MDSC) and Suppress Microbead or Lectin-Induced T Cell Proliferation Through Artefactual Mechanisms. Sci Rep (2018) 8:3135. doi: 10.1038/s41598-018-21450-6 29453429 PMC5816646

[12] Zhou J Nefedova Y Lei A Gabrilovich D Neutrophils and PMN-MDSC. Their Biological Role and Interaction With Stromal Cells. Semin Immunol (2018) 35:19–28. doi: 10.1016/j.smim.2017.12.004 29254756 PMC5866202

[13] Tcyganov E Mastio J Chen E Gabrilovich DI. Plasticity of Myeloid-Derived Suppressor Cells in Cancer. Curr Opin Immunol (2018) 51:76–82. doi: 10.1016/j.coi.2018.03.009 29547768 PMC5943174

[14] van der Touw W Chen HM Pan PY Chen SH. LILRB Receptor-Mediated Regulation of Myeloid Cell Maturation and Function. Cancer Immunol Immunother (2017) 66:1079–87. doi: 10.1007/s00262-017-2023-x PMC5591173 28638976

[15] Zhang J Mai S Chen HM Kang K Li XC Chen SH. Leukocyte Immunoglobulin-Like Receptors in Human Diseases: An Overview of Their Distribution, Function, and Potential Application for Immunotherapies. J Leukoc Biol (2017) 102:351–60. doi: 10.1189/jlb.5MR1216-534R PMC5505746 28351852

[16] Chen HM van der Touw W Wang YS Kang K Mai S Zhang J. Blocking Immunoinhibitory Receptor LILRB2 Reprograms Tumor-Associated Myeloid Cells and Promotes Antitumor Immunity. J Clin Invest (2018) 128:5647–62. doi: 10.1172/JCI97570 PMC6264729 30352428

[17] Lechner MG Liebertz DJ Epstein AL. Characterization of Cytokine-Induced Myeloid-Derived Suppressor Cells From Normal Human Peripheral Blood Mononuclear Cells. J Immunol (2010) 185:2273–84. doi: 10.4049/jimmunol.1000901 PMC2923483 20644162

[18] Jagannath C Lindsey DR Dhandayuthapani S Xu Y Hunter RL Jr. Eissa NT. Autophagy Enhances the Efficacy of BCG Vaccine by Increasing Peptide Presentation in Mouse Dendritic Cells. Nat Med (2009) 15:267–76. doi: 10.1038/nm.1928 19252503

[19] Jagannath C Actor JK Hunter RL Jr. Induction of Nitric Oxide in Human Monocytes and Monocyte Cell Lines by Mycobacterium Tuberculosis. Nitric Oxide (1998) 2:174–86. doi: 10.1006/niox.1998.9999 9731635

[20] Daniel DS Dai G Singh CR Lindsey DR Smith AK Dhandayuthapani S. The Reduced Bactericidal Function of Complement C5-Deficient Murine Macrophages is Associated With Defects in the Synthesis and Delivery of Reactive Oxygen Radicals to Mycobacterial Phagosomes. J Immunol (2006) 177:4688–98. doi: 10.4049/jimmunol.177.7.4688 16982908

[21] Khan A Mann L Papanna R Lyu MA Singh CR Olson S. Mesenchymal Stem Cells Internalize Mycobacterium Tuberculosis Through Scavenger Receptors and Restrict Bacterial Growth Through Autophagy. Sci Rep (2017) 7:15010. doi: 10.1038/s41598-017-15290-z 29118429 PMC5678154

[22] Ramakrishnan L. Revisiting the Role of the Granuloma in Tuberculosis. Nat Rev Immunol (2012) 12:352–66. doi: 10.1038/nri3211 22517424

[23] Refai A Gritli S Barbouche MR Essafi M. Mycobacterium Tuberculosis Virulent Factor ESAT-6 Drives Macrophage Differentiation Toward the Pro-Inflammatory M1 Phenotype and Subsequently Switches It to the Anti-Inflammatory M2 Phenotype. Front Cell Infect Microbiol (2018) 8:327. doi: 10.3389/fcimb.2018.00327 30283745 PMC6157333

[24] Tripal P Bauer M Naschberger E Mortinger T Hohenadl C Cornali E. Unique Features of Different Members of the Human Guanylate-Binding Protein Family. J Interferon Cytokine Res (2007) 27:44–52. doi: 10.1089/jir.2007.0086 17266443

[25] Kim BH Shenoy AR Kumar P Das R Tiwari S MacMicking JD. A Family of IFN-Gamma-Inducible 65-kD GTPases Protects Against Bacterial Infection. Science (2011) 332:717–21. doi: 10.1126/science.1201711 21551061

[26] Marinho FV Fahel JS de Araujo A Diniz LTS Gomes MTR Resende DP. Guanylate Binding Proteins Contained in the Murine Chromosome 3 Are Important to Control Mycobacterial Infection. J Leukoc Biol (2020) 108:1279–91. doi: 10.1002/JLB.4MA0620-526RR 32620042

[27] Blanchette J Abu-Dayyeh I Hassani K Whitcombe L Olivier M. Regulation of Macrophage Nitric Oxide Production by the Protein Tyrosine Phosphatase Src Homology 2 Domain Phosphotyrosine Phosphatase 1 (SHP-1). Immunology (2009) 127:123–33. doi: 10.1111/j.1365-2567.2008.02929.x PMC2678188 18793215

[28] Langemeyer L Frohlich F Ungermann C. Rab GTPase Function in Endosome and Lysosome Biogenesis. Trends Cell Biol (2018) 28:957–70. doi: 10.1016/j.tcb.2018.06.007 30025982

[29] Yu L Chen Y Tooze SA. Autophagy Pathway: Cellular and Molecular Mechanisms. Autophagy (2018) 14:207–15. doi: 10.1080/15548627.2017.1378838 PMC5902171 28933638

[30] Upadhyay S Philips JA. LC3-Associated Phagocytosis: Host Defense and Microbial Response. Curr Opin Immunol (2019) 60:81–90. doi: 10.1016/j.coi.2019.04.012 31247378 PMC12154396

[31] Zulauf KE Sullivan JT Braunstein M. The SecA2 Pathway of Mycobacterium Tuberculosis Exports Effectors That Work in Concert to Arrest Phagosome and Autophagosome Maturation. PloS Pathog (2018) 14:e1007011. doi: 10.1371/journal.ppat.1007011 29709019 PMC5945054

[32] Saikolappan S Estrella J Sasindran SJ Khan A Armitige LY Jagannath C. The Fbpa/sapM Double Knock Out Strain of Mycobacterium Tuberculosis is Highly Attenuated and Immunogenic in Macrophages. PloS One (2012) 7:e36198. doi: 10.1371/journal.pone.0036198 22574140 PMC3344844

[33] Martinez FO Gordon S. The M1 and M2 Paradigm of Macrophage Activation: Time for Reassessment. F1000Prime Rep (2014) 6:13. doi: 10.12703/P6-13 24669294 PMC3944738

[34] Chistiakov DA Myasoedova VA Revin VV Orekhov AN Bobryshev YV. The Impact of Interferon-Regulatory Factors to Macrophage Differentiation and Polarization Into M1 and M2. Immunobiology (2018) 223:101–11. doi: 10.1016/j.imbio.2017.10.005 29032836

[35] Master SS Rampini SK Davis AS Keller C Ehlers S Springer B. Mycobacterium Tuberculosis Prevents Inflammasome Activation. Cell Host Microbe (2008) 3:224–32. doi: 10.1016/j.chom.2008.03.003 PMC3657562 18407066

[36] Schick J Schafer J Alexander C Dichtl S Murray PJ Christensen D. Cutting Edge: TNF Is Essential for Mycobacteria-Induced MINCLE Expression, Macrophage Activation, and Th17 Adjuvanticity. J Immunol (2020) 205:323–8. doi: 10.4049/jimmunol.2000420 32540999

[37] Agrawal N Streata I Pei G Weiner J Kotze L Bandermann S. Human Monocytic Suppressive Cells Promote Replication of Mycobacterium Tuberculosis and Alter Stability of *In Vitro* Generated Granulomas. Front Immunol (2018) 9:2417. doi: 10.3389/fimmu.2018.02417 30405617 PMC6205994

[38] du Plessis N Kotze LA Leukes V Walzl G. Translational Potential of Therapeutics Targeting Regulatory Myeloid Cells in Tuberculosis. Front Cell Infect Microbiol (2018) 8:332. doi: 10.3389/fcimb.2018.00332 30298121 PMC6160538

[39] Bernard EM Fearns A Bussi C Santucci P Peddie CJ Lai RJ. M. Tuberculosis Infection of Human iPSC-Derived Macrophages Reveals Complex Membrane Dynamics During Xenophagy Evasion. J Cell Sci 134 (2020) 134(5):jcs252973. doi: 10.1242/jcs.252973 PMC7710011 32938685

[40] Fletcher M Ramirez ME Sierra RA Raber P Thevenot P Al-Khami AA. L-Arginine Depletion Blunts Antitumor T-Cell Responses by Inducing Myeloid-Derived Suppressor Cells. Cancer Res (2015) 75:275–83. doi: 10.1158/0008-5472.CAN-14-1491 PMC4297565 25406192

[41] Yu J Du W Yan F Wang Y Li H Cao S. Myeloid-Derived Suppressor Cells Suppress Antitumor Immune Responses Through IDO Expression and Correlate With Lymph Node Metastasis in Patients With Breast Cancer. J Immunol (2013) 190:3783–97. doi: 10.4049/jimmunol.1201449 23440412

[42] El Daker S Sacchi A Tempestilli M Carducci C Goletti D Vanini V. Granulocytic Myeloid Derived Suppressor Cells Expansion During Active Pulmonary Tuberculosis is Associated With High Nitric Oxide Plasma Level. PloS One (2015) 10:e0123772. doi: 10.1371/journal.pone.0123772 25879532 PMC4400140

[43] Reis-Sobreiro M Teixeira da Mota A Jardim C Serre K. Bringing Macrophages to the Frontline Against Cancer: Current Immunotherapies Targeting Macrophages. Cells 10 (2021) 10(9):2364. doi: 10.3390/cells10092364 PMC8464913 34572013

[44] Yang S Wei Y Sun R Lu W Lv H Xiao X. Umbilical Cord Blood-Derived Mesenchymal Stromal Cells Promote Myeloid-Derived Suppressor Cell Proliferation by Secreting HLA-G to Reduce Acute Graft-Versus-Host Disease After Hematopoietic Stem Cell Transplantation. Cytotherapy (2020) 22:718–33. doi: 10.1016/j.jcyt.2020.07.008 32811747

[45] Zhao Y van Woudenbergh E Zhu J Heck AJR van Kessel KPM de Haas CJC. The Orphan Immune Receptor LILRB3 Modulates Fc Receptor-Mediated Functions of Neutrophils. J Immunol (2020) 204:954–66. doi: 10.4049/jimmunol.1900852 PMC7617070 31915259

[46] Vinod V Vijayrajratnam S Vasudevan AK Biswas R. The Cell Surface Adhesins of Mycobacterium Tuberculosis. Microbiol Res (2020) 232:126392. doi: 10.1016/j.micres.2019.126392 31841935

[47] Aderem A Underhill DM. Mechanisms of Phagocytosis in Macrophages. Annu Rev Immunol (1999) 17:593–623. doi: 10.1146/annurev.immunol.17.1.593 10358769

[48] Yao K Chen Q Wu Y Liu F Chen X Zhang Y. Unphosphorylated STAT1 Represses Apoptosis in Macrophages During Mycobacteriumtuberculosis Infection. J Cell Sci (2017) 130:1740–51. doi: 10.1242/jcs.200659 28348106

[49] Yang CS Lee JS Song CH Hur GM Lee SJ Tanaka S. Protein Kinase C Zeta Plays an Essential Role for Mycobacterium Tuberculosis-Induced Extracellular Signal-Regulated Kinase 1/2 Activation in Monocytes/Macrophages *via* Toll-Like Receptor 2. Cell Microbiol (2007) 9:382–96. doi: 10.1111/j.1462-5822.2006.00797.x 16925784

[50] Seto S Tsujimura K Koide Y. Coronin-1a Inhibits Autophagosome Formation Around Mycobacterium Tuberculosis-Containing Phagosomes and Assists Mycobacterial Survival in Macrophages. Cell Microbiol (2012) 14:710–27. doi: 10.1111/j.1462-5822.2012.01754.x 22256790

[51] Dempke WCM Uciechowski P Fenchel K Chevassut T SHP-1 T. 2 and SHIP Pathways: A Novel Strategy for Cancer Treatment? Oncology (2018) 95:257–69. doi: 10.1159/000490106 29925063

[52] Raghuvanshi S Sharma P Singh S Van Kaer L Das G. Mycobacterium Tuberculosis Evades Host Immunity by Recruiting Mesenchymal Stem Cells. Proc Natl Acad Sci U.S.A. (2010) 107:21653–8. doi: 10.1073/pnas.1007967107 PMC3003090 21135221

[53] Khan A Hunter RL Jagannath C. Emerging Role of Mesenchymal Stem Cells During Tuberculosis: The Fifth Element in Cell Mediated Immunity. Tuberc (Edinb) (2016) 101S:S45–52. doi: 10.1016/j.tube.2016.09.019 27743705

