# Supplementary material for: Antibody-Mediated LILRB2-Receptor Antagonism Induces Human Myeloid-Derived Suppressor Cells to Kill Mycobacterium tuberculosis
Source: Front Immunol. 2022 Jun 10;13:865503. doi: 10.3389/fimmu.2022.865503 (PMC9229593; doi:10.3389/fimmu.2022.865503)
Supplement: Supplementary file 1 [file Image_1.pdf]

Supplementary Figure 1. Flow cytometry to check the purification of Monocytic (Mo) MDSCs

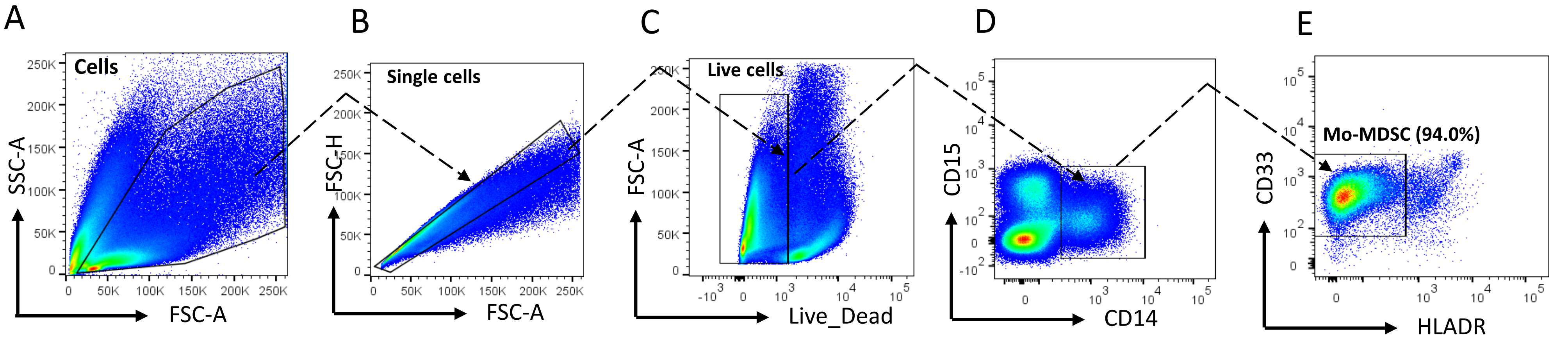

<u>Human Monocytic (Mo) MDSC</u>: $CD15^{-}CD14^{+}CD33^{+}HLADR^{-}$
