# Supplementary material for: Antibody-Mediated LILRB2-Receptor Antagonism Induces Human Myeloid-Derived Suppressor Cells to Kill Mycobacterium tuberculosis
Source: Front Immunol. 2022 Jun 10;13:865503. doi: 10.3389/fimmu.2022.865503 (PMC9229593; doi:10.3389/fimmu.2022.865503)
Supplement: Supplementary file 2 [file Image_2.pdf]

Supplementary Figure 2

'Protein Simple' Western Assay / Wes analysis of proteins in lysates of Mtb infected cells. For the analysis of protein levels of macrophages/MDSC, we used the quantitative Wes capillary immunoassay, in which proteins were separated and detected using Wes separation capillary cartridge 12-230 kDa along with Wes Anti-Rabbit Detection Module (Simple Western system and Compass Software, Protein Simple). In brief, glass microcapillaries were loaded with stacking and separation matrices followed by sample loading. During capillary electrophoresis, proteins were separated by size and then immobilized to the capillary wall. Samples were loaded at 1 mg/ml dilution and the primary rabbit antibodies and GAPDH were used at 1:50 dilution. Data were analyzed with the Compass software (version 2.6.7). The area under the curve (AUC), which represents the signal intensity of the chemiluminescent reaction was analyzed for all the antibodies and GAPDH. Values given for protein expression were normalized to GAPDH. Quantitation of protein levels (area under each peak; arbitrary units [A.U.]) were performed using the Compass software (version 2.6.7). An example is shown here.

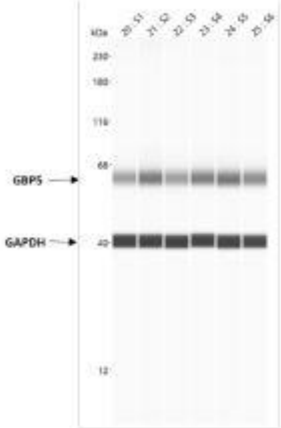

A. Original Automated Wes Western blot showing GBP5 levels on the top and GAPDH on the bottom

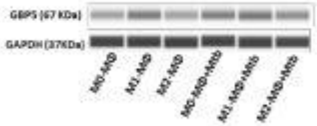

B. Automated Wes Western blot showing GBP5 and GAPDH levels edited from (A)

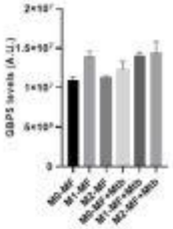

C. Quantitation of GBP5 levels (area under each peak; arbitrary units [A.U.]

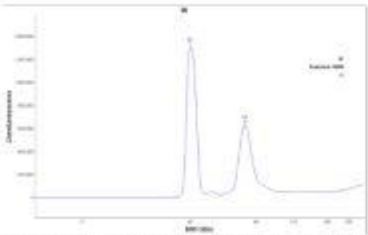

D. Electropherogram showing the expression level of GBP5 and GAPDH (Sample M0-MΦ)

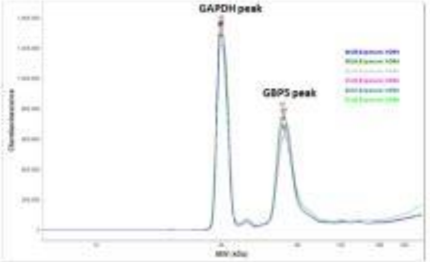

E. Overlay (sample 1-6) of electropherograms displayed in (D)
