# Supplementary material for: Antibody-Mediated LILRB2-Receptor Antagonism Induces Human Myeloid-Derived Suppressor Cells to Kill Mycobacterium tuberculosis
Source: Front Immunol. 2022 Jun 10;13:865503. doi: 10.3389/fimmu.2022.865503 (PMC9229593; doi:10.3389/fimmu.2022.865503)

Supplementary Figure 3

Myeloid Populations

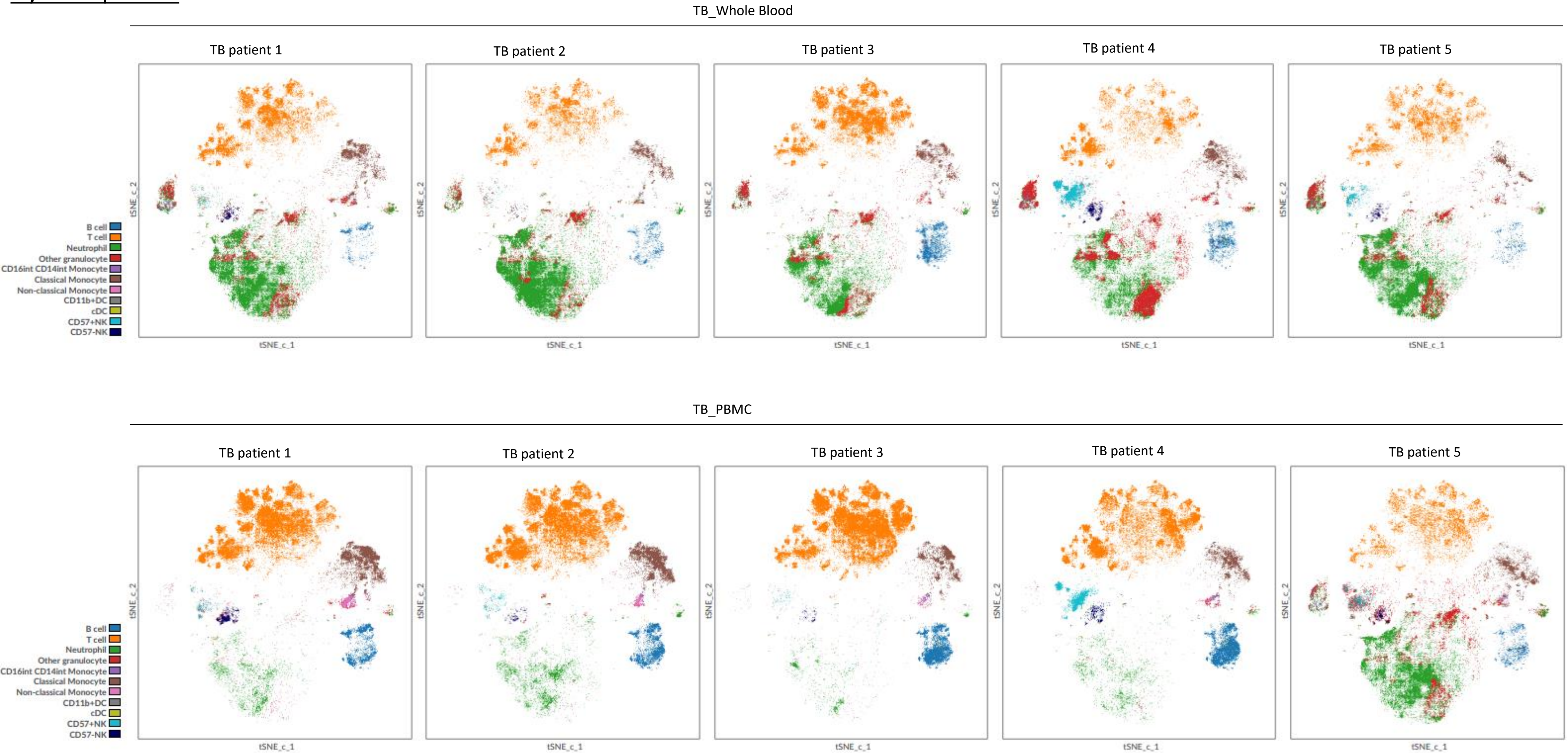

Myeloid Populations

Healthy Control_Whole Blood

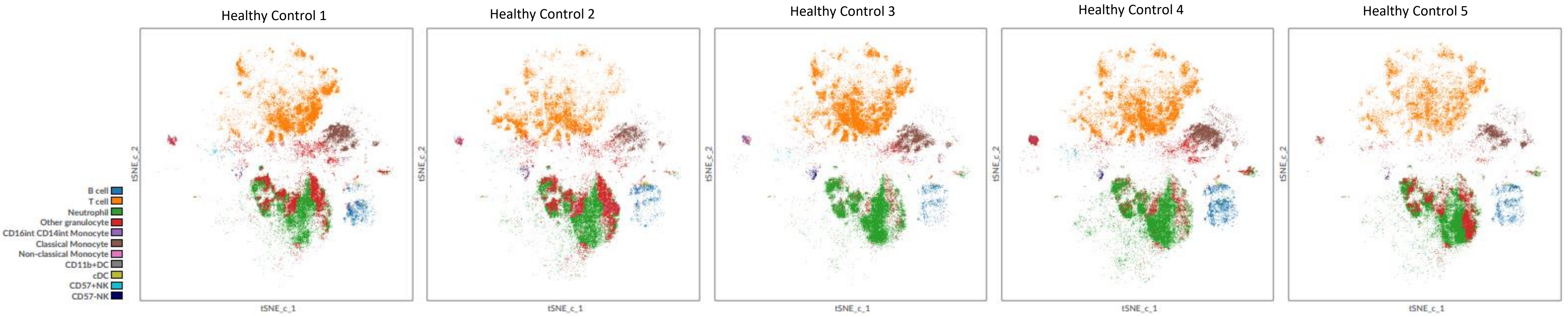

Healthy Control_PBMC

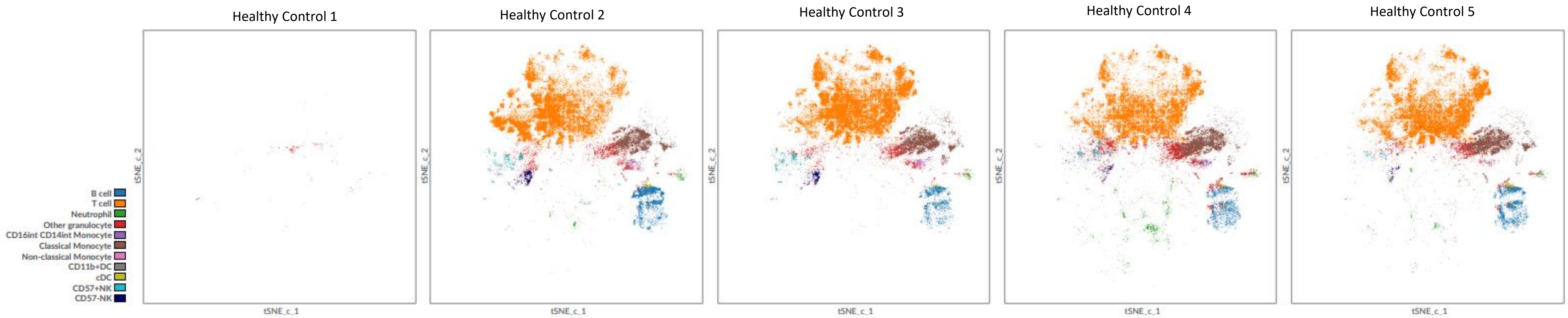

Lymphoid Populations

TB_Whole Blood

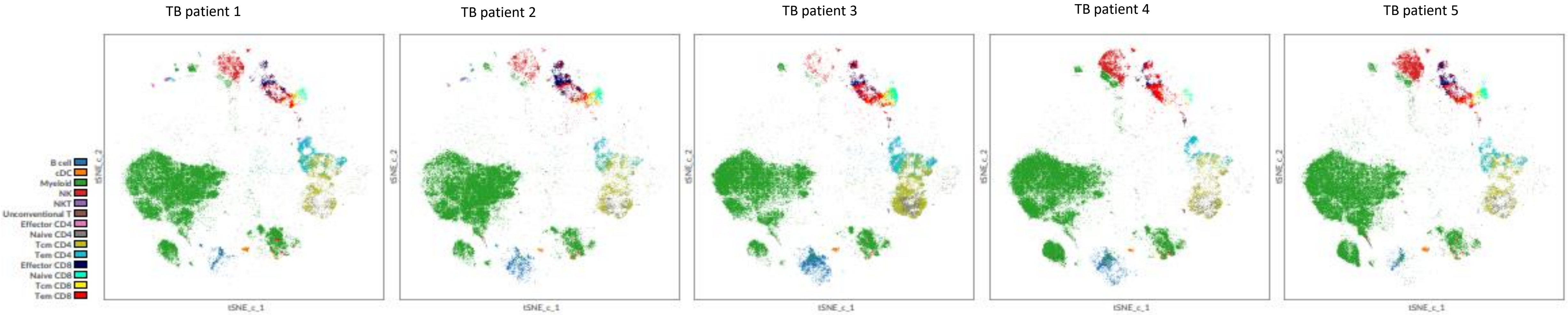

TB_PBMC

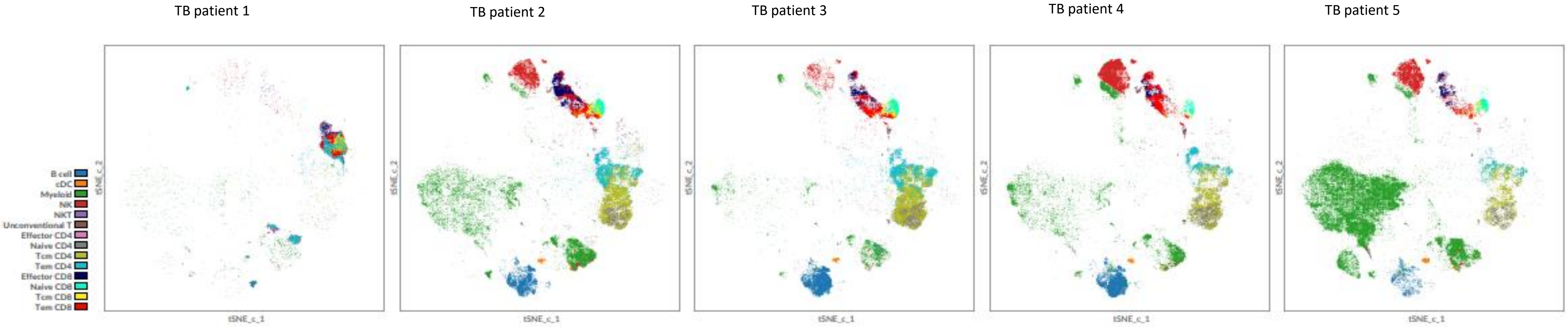

Lymphoid Populations

Healthy Control_Whole Blood

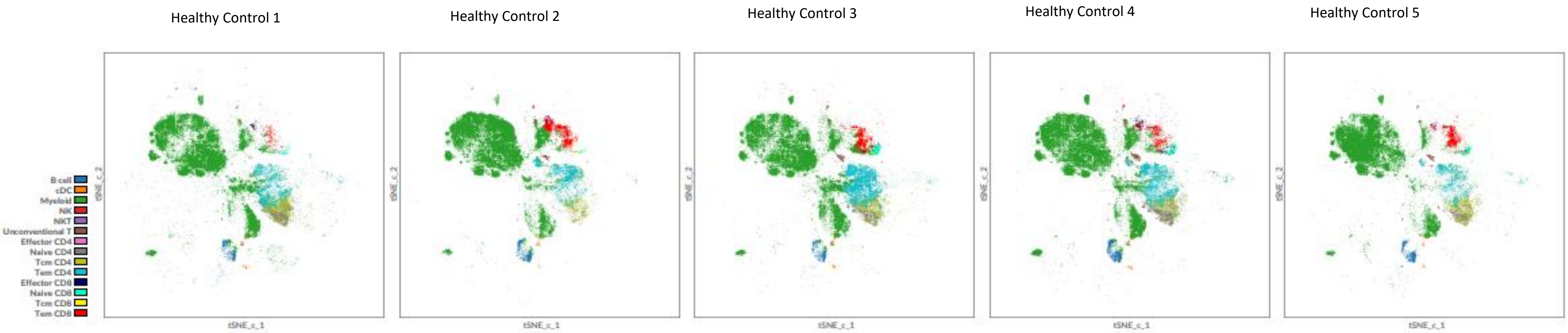

Healthy Control_PBMC

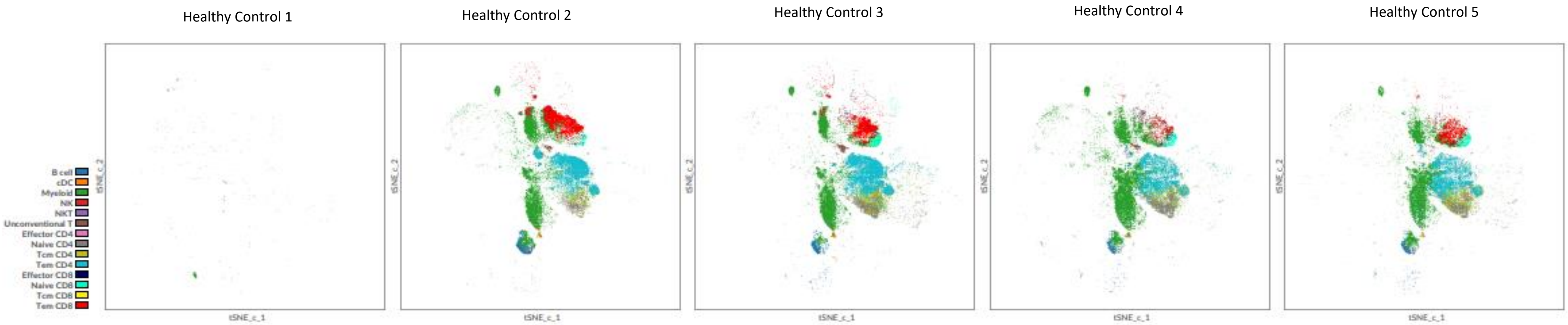

Supplement: Supplementary file 3 [file Image_3.pdf]
